# Pulmonary delivery of influenza vaccine formulations in cotton rats: site of deposition plays a minor role in the protective efficacy against clinical isolate of H1N1pdm virus

**DOI:** 10.1080/10717544.2018.1435748

**Published:** 2018-02-16

**Authors:** Yoshita Bhide, Jasmine Tomar, Wei Dong, Jacqueline de Vries-Idema, Henderik W. Frijlink, Anke Huckriede, Wouter L. J. Hinrichs

**Affiliations:** a Department of Medical Microbiology, University of Groningen, University Medical Center Groningen, Groningen, The Netherlands;; b Department of Pharmaceutical Technology and Biopharmacy, University of Groningen, Groningen, The Netherlands

**Keywords:** Whole inactivated influenza virus vaccine, inhalation, deposition, immunogenicity, protection

## Abstract

Administration of influenza vaccines to the lungs could be an attractive alternative to conventional parenteral administration. In this study, we investigated the deposition site of pulmonary delivered liquid and powder influenza vaccine formulations and its relation to their immunogenicity and protective efficacy. *In vivo* deposition studies in cotton rats revealed that, the powder formulation was mainly deposited in the trachea ( ∼ 65%) whereas the liquid was homogenously distributed throughout the lungs ( ∼ 96%). In addition, only 60% of the antigen in the powder formulation was deposited in the respiratory tract with respect to the liquid formulation. Immunogenicity studies showed that pulmonary delivered liquid and powder influenza formulations induced robust systemic and mucosal immune responses (significantly higher by liquids than by powders). When challenged with a clinical isolate of homologous H1N1pdm virus, all animals pulmonary administered with placebo had detectable virus in their lungs one day post challenge. In contrast, none of the vaccinated animals had detectable lung virus titers, except for two out of eight animals from the powder immunized group. Also, pulmonary vaccinated animals showed no or little signs of infection like increase in breathing frequency or weight loss upon challenge as compared to animals from the negative control group. In conclusion, immune responses induced by liquid formulation were significantly higher than responses induced by powder formulation, but the overall protective efficacy of both formulations was comparable. Thus, pulmonary immunization is capable of inducing protective immunity and the site of antigen deposition seems to be of minor relevance in inducing protection.

## Introduction

Influenza is one of the major respiratory diseases with high morbidity and mortality (WHO, 2016; Kondrich, 2017). Annually, influenza leads to 3–5 million hospitalizations and around 250,000–500,000 deaths worldwide (WHO, 2016). It is generally recognized that vaccination is the best strategy to prevent the disease. Currently, most influenza vaccines are administered via intramuscular injection (i.m.), which can induce systemic but very little or no mucosal immune responses (Cox et al., 2004; Amorij et al., 2007). Influenza virus is mainly transmitted via the airways and replicates in the respiratory epithelium, thus curbing the infection at the portal of entry would be a beneficial approach (Brandtzaeg, 2007; van Riet et al., 2012). In addition, mucosal areas such as lungs are excellent targets for influenza immunization because of their large surface area, which is loaded with a large number of antigen presenting cells (Amorij et al., 2007; Vareille et al., 2011).

Targeting lungs for influenza vaccination commenced in 1960’s. It was found that liquid formulation pulmonary administered to human subjects was as effective as the traditional i.m. administered influenza vaccine in preventing illness (Waldman et al., 1969a,b; Haigh & Howell, 1973). Most likely due to the complicated and laborious use of nebulizers available at that time, these studies were discontinued and pulmonary targeting was not investigated for a long time (Saluja et al., 2010; Murugappan et al., 2013). Recently, the pulmonary route has regained attention in preclinical influenza vaccine research using appropriate animal models (Amorij et al., 2007; Wee et al., 2008; Peeters et al., 2014; Sou et al., 2015). Influenza vaccines for pulmonary administration can be formulated as liquids and as well as dry powders (Tomar et al., 2016).

Commonly, liquid formulations can be pulmonary administered to animals using the Penn-Century MicroSprayer (Wyndmoor, PA). When enough liquid is administered using this device, whole lung deposition can be expected as liquids can drip down. For dry powder formulations, it is well known that an aerodynamic particle size of 1–5 µm is required in order to achieve antigen deposition in the whole lung (Cheng, 2014; Sheth et al., 2015). Our previous studies have shown that particles of this size distribution can be prepared by spray freeze drying (SFD) (Amorij et al., 2007; Saluja et al., 2010; Audouy et al., 2011; Murugappan et al., 2013; Patil et al., 2017). However, we found that one of the most frequently used device that was available for pulmonary administration of dry powders (Penn-century insufflator) to experimental animals cannot efficiently de-agglomerate SFD powder particles (Murugappan et al., 2013; Hoppentocht et al., 2014). Accordingly, the particle agglomerates as dispersed by this device are relatively large and as such are expected to be deposited in the upper airways instead of the whole lung. Hence we made use of this drawback of the insufflator to achieve high lung deposition. In some pre-clinical studies, for vaccine candidates against tuberculosis and also against influenza, deep lung targeting has already shown an edge over targeting upper parts of the respiratory tract (Minne et al., 2007; Todoroff et al., 2013). In the study by Minne et al. (2007) better immune responses were found to be elicited when liquid influenza vaccine was targeted to the deep lung instead of the upper or central airways. However, the deposition site of powder formulations and its possible effect on immune responses was not determined. Also, it was not investigated whether or not the site of deposition in the respiratory tract is of relevance for the protective efficacy of vaccine formulations.

Majority of the studies on pulmonary administration of influenza vaccines have been done in the commonly used animal model for influenza research i.e. mice. In contrast to mice which usually require the use of mouse adapted influenza virus strains for challenge (Bouvier & Lowen, 2010), cotton rats are susceptible to infection by unadapted, human influenza virus strains (Eichelberger et al., 2004; Ottolini et al., 2005; Blanco et al., 2014). Disease progression in cotton rats is symptomatic and can be frequently monitored by measuring breathing frequency, weight loss, and temperature drop (Eichelberger et al., 2004; Ottolini et al., 2005; Blanco et al., 2013). Furthermore, compared to mice, the larger size of cotton rats makes them less prone to potential mechanical damage during pulmonary delivery.

The aim of the current study was to investigate whether a) cotton rats can be used as a model for influenza vaccination via the pulmonary route; b) difference in the site of deposition of liquid and dry influenza vaccine formulations has an impact on the immunogenicity and thus the protective efficacy of these formulations. Our results indicate that pulmonary delivery can successfully be done in cotton rats, with liquid and dry powder influenza vaccine formulations being deposited in different parts of the respiratory tract. Further, both liquid and powder influenza formulations had the potential to induce protection upon challenge with a clinically relevant virus strain.

## Materials and methods

### Virus and vaccine

NIBRG-121, a vaccine strain derived from A/California/7/2009 H1N1pdm09 virus obtained from NIBSC (Potters Bay, UK), was grown on embryonated chicken eggs as described previously (Audouy et al., 2011). The virus was inactivated by overnight treatment with 0.1% β-propiolactone (Acros Organics, Geel, Belgium) in citrate buffer (125 mM sodium citrate, 150 mM sodium chloride, pH 8.2) at 4 °C to produce whole inactivated influenza virus vaccine (WIV). After inactivation, WIV was dialyzed against HNE buffer (145 mM NaCl, 5 mM Hepes, 1 mM EDTA, pH 7.4, and sterilized by autoclaving) to completely remove β-propiolactone. Inactivation was verified by inoculating WIV with Madin-Darby Canine Kidney (MDCK) cells and the readout was done by hemagglutination assay as described before (Audouy et al., 2011). The protein concentration of the obtained WIV preparation was determined by micro-Lowry assay. The vaccine dose was based on hemagglutinin (HA) content which was assumed to be 1/3rd of the total viral protein weight as described previously (Patil et al., 2014).

For the challenge study, a clinical isolate of A/California/2009 (E9-6714) provided by the Department of Clinical Virology, UMCG, Groningen, The Netherlands was used. This virus was grown in MDCK cells and titrated in cotton rats. This virus will be termed as A/Cal/2009 in the following sections.

### Labeling of whole inactivated influenza virus vaccine

WIV was labeled with the near infrared fluorescent dye VivoTag 680XL (Perkin Elmer, Waltham, MA) as per the manufacturer’s protocol. Briefly, 3.3 mL of vaccine solution (concentration: 0.31 mg/mL of HA) was mixed with 6.5 µL of VivoTag 680XL (concentration: 25 µg/µL) and 220 µL of 1 M sodium bi carbonate (NaHCO_3_) solution. The mixture was incubated at room temperature for two hours under constant shaking. Thereafter, the unbound fluorophore was removed by using Zeba Spin Desalting Columns (ThermoScientific, Rockford, IL). The degree of labeling was calculated by determining the labeled WIV and the dye concentration at 280 and 668 nm, respectively. To adjust for fluorophore crosstalk at 280 nm, 16% of the absorbance at 668 nm was subtracted from the absorbance at 280 nm. The degree of labeling was found to be approximately two, which means that on average each WIV particle was labeled with two dye molecules.

### Preparation of the powders by SFD

Labeled or unlabeled WIV was spray freeze dried together with inulin (4 kDa, Sensus, Roosendaal, The Netherlands) as lyoprotectant. Inulin powders were prepared (from inulin solution in water) without influenza vaccine using the similar procedure. Briefly, labeled and unlabeled vaccine solutions were prepared in an HA: inulin weight ratio of 1:200 and 1:40, respectively. The HA: inulin weight ratios of 1:200 and 1:40 were based on a dose of 5 µg (deposition study) and 25 µg HA (immunogenicity study) in 1 mg of SFD powder. The vaccine solutions were sprayed into a vessel of liquid nitrogen using the two-fluid nozzle of the Buchi 190 Mini Spray Dryer with an inner diameter of 0.5 mm. The nozzle was placed approximately 5 cm above the level of liquid nitrogen. A liquid flow rate of 5 mL/min and an atomizing airflow of 600 L_n_/h were used. Then, the frozen vaccine solutions were freeze dried for 48 h under the following conditions: during the first 24 h the shelf temperature was set at −35 °C and the pressure at 0.220 mbar, after which the, temperature was gradually increased to 20 °C and the pressure was lowered to 0.05 mbar during the next 24 h. The spray freeze dried vaccine formulations were collected in a chamber with a relative humidity ≤1% and ambient temperature. Until further use, the obtained powders were stored under airtight conditions.

### Characterization of vaccine powders

#### Particle size analysis

Laser diffraction was used for determining the primary particle size distribution of spray freeze dried powders. RODOS (Sympatec, Clausthal-Zellerfeld Germany) was used as the disperser and a pressure of 1 bar was applied for the dispersion of the powder. A 100 nm (R3) lens was used. For particle size measurements with the Penn-Century insufflator (Penn-Century, Wyndmoor, PA), the tip of the insufflator and microsprayer was kept at a constant distance from the laser beam by mounting them on an in-house mounting plate. For insufflator, an AP-1 air pump was used to deliver 1 mL of air. The geometric particle size distributions were calculated according to the Fraunhofer theory.

#### Scanning electron microscopy (SEM)

A Jeol JSM 6301-F microscope was used for scanning electron microscopy. Powders were placed on a double sided sticky carbon tape on a metal disc. Then, the particles were coated with 30 nm of gold using a Balzer’s 120B sputtering device (Balzer, Union, Austria). Images were taken at a magnification of 500× and 5000×.

### Hemagglutination assay

The receptor binding activity of WIV after spray freeze drying (unlabeled WIV formulation) was assessed by the hemagglutination assay as described previously (Audouy et al., 2011). Briefly, WIV was reconstituted in Phosphate buffered saline (PBS) and 50 µL was added to 96 V bottom plates containing 50 µl of PBS. Two fold serial dilutions were prepared after which 50 µL of 1% guinea pig red blood cells suspension was added to each well. Hemagglutination titers were read two hours after incubation at room temperature and are expressed as log_2_ of the highest dilution where red blood cells (RBC) agglutination could be seen.

### In vivo *experiments*

All animal experiments were approved by the Institutional Animal Care and Use Committee of the University of Groningen (IACUC-RUG), The Netherlands. Outbred female cotton rats at an age of 10–12 weeks were purchased from Envigo, Somerset, NJ.. Animals were housed as two animals per cage and were given standard diet and water.

#### *Deposition study and* in-vivo *imaging system* (*IVIS) measurements*

Cotton rats were anesthetized using isoflurane and the anesthetized animals were brought to vertical position and intubated with an Autograde catheter (14 G, BD, Breda, The Netherlands). 50 µL of liquid vaccine containing 5 µg HA was delivered through the catheter to six cotton rats using IA-1 C-R microsprayer attached to a FMJ 250 high pressure syringe (Penn-Century, Wyndmoor, RA). For powder formulations, a customized length Penn-Century insufflator was used to deliver 1 mg of spray freeze dried powder containing 5 µg HA (*n* = 6). Three puffs of 1 mL air were used for the dispersion of the powder. The tip of the dispersion device was placed just above the carina of the animals. Immediately after vaccine administration, cotton rats were sacrificed and lungs along with trachea were taken out. Lungs either as such or dissected into lung lobes were placed in petri dishes and evaluated by an IVIS^®^ Spectrum (Perkin Elmer, Waltham, MA). Excitation wavelength of 675 nm was used to measure the fluorescent emission at 720 nm. The intensity of the emitted light (photons/s/cm^2^/steradian) was quantified using Living Image Software v3.2 (Perkin Elmer). Fluorescent intensities of the vaccinated animals were corrected by subtracting the fluorescent intensities of untreated animals (*n* = 3).

#### Immunization and challenge study

For this experiment, animals were injected with implantable electronic ID transponders via subcutaneous (s.c.) route for identification. Weights of the cotton rats around challenge phase ranged between 120–150 g. Cotton rats were vaccinated via the pulmonary route with influenza WIV liquid [WIV (Pul-Liq), *n* = 11] or 1 mg powder [WIV (Pul-Pow), *n* = 11] formulations with an antigen dose of 25 µg HA for both liquid and powder formulations. Cotton rats were intubated and influenza vaccine was administered similarly as described for the *in vivo* deposition study. As a gold standard, 100 µL of WIV containing 5 µg HA in HNE buffer was administered via the intramuscular (i.m.) injection, with 50 µL divided over both hind limbs of the animals (WIV i.m., *n* = 9). Animals pulmonary administered with 1 mg SFD inulin alone were used as negative control [inulin (Pul-Pow), *n* = 9]. Animals were vaccinated twice with an interval of three weeks between the two vaccinations. Before challenge, three animals of the pulmonary liquid group died due to unknown reasons. Three weeks after the second vaccination, all cotton rats were challenged intranasally (i.n.) with 1 × 10^7^ TCID50 of A/Cal/2009 virus in 100 µL dose volume distributed over both nostrils using a pipette. The chosen dose volume was 100 µL dose volume in order to cover the whole respiratory tract (Southam et al., 2002). Both vaccination and challenge were carried out under 5% isoflurane/oxygen anesthesia.

### Sample collection

On the day of challenge, blood was drawn from the animals by orbital puncture and serum was separated to evaluate humoral immune responses. One day post challenge animals were sacrificed [*n* = 5 for WIV (Pul-Liq), *n* = 8 for WIV (Pul-Pow), *n* = 6 for WIV (i.m.), and *n* = 6 for inulin (Pul-Pow)]. After sacrifice, nasal and lung washes were collected in 1 mL PBS containing Complete^®^ protease inhibitor cocktail tablets (Roche, Almere, The Netherlands) through a hole made in the trachea, for the determination of antibody and MN titers. For virus titration, lungs were collected in a pre-weighed tube containing complete EPISERF medium (100 U/ml penicillin, 100 mg/ml streptomycin, 1 M HEPES, and 7.5% sodium bicarbonate, all Life Technologies^TM^ BV, Bleiswijk, The Netherlands). The tubes with lungs were weighed again and weight of the lung was calculated.

### Systemic and mucosal immune responses

Immunoglobin G (IgG) titers were evaluated by ELISA in serum collected on the day of challenge and in the lung washes. IgA antibodies were determined in lung and nasal washes collected from the animals sacrificed one day post challenge. ELISA was performed as described previously (Liu et al., 2012). IgA and IgG titers were calculated as log_10_ of the reciprocal of the sample dilution corresponding to an absorbance of 0.2 at the wavelength of 492 nm. As described before, the functional potential of systemic antibodies was assessed by microneutralization (MN) and hemagglutination inhibition (HI) assay using serum samples taken on the challenge day (Budimir et al., 2012; Liu et al., 2012). MN titers were also determined in lung washes taken one day post challenge. MN titers are presented as log_2_ titers for individual cotton rats for serum MN and pooled for lung MN. For HI sera were pooled per group and also for MN lung lavages were pooled per group, as these samples were not enough to perform these assays using individual sample per animal. HI titers are presented as log_2_ for pooled serum per group. Limit of detection (LoD) for IgG titers was determined by calculating log_10_ of the first dilution made and the negative control samples were given a value corresponding to half of the LoD for calculation purposes. LoD for MN and HI was calculated in a similar way considering log_2_.

#### Lung virus titration

Lung virus titers were determined using lung homogenates collected one day post challenge as described previously (Audouy et al., 2011). Briefly, lung homogenates were serially diluted two fold using complete EpiSerf medium and inoculated with MDCK cells. After one hour the medium was replaced with EpiSerf containing 5 µg/mL L-1-Tosylamide-2-phenylethyl chloromethyl ketone (TPCK) trypsin and incubated for 72 h at 37 °C, 5% CO_2_. Viral titers were determined by adding 1% guinea pig RBC to cell supernatants and scoring hemagglutination. Virus titers are depicted as log_10_ lung virus titers per gram of lung. Limit of detection (LoD) was determined by calculating log_10_ of the first dilution made and the negative control samples were given the value as log_10_ of half value of the first dilution.

### Assessment of clinical symptoms

Clinical symptoms were assessed daily for ten days after virus challenge. Briefly, upon challenge, remaining animals (*n* = 3 for all groups) were followed daily to assess changes in weight, temperature, and breathing frequency (BF) for 10 days. The animals restrained in a pre-weighed cardboard rolls were weighed and by subtracting the weight of the roll, the weight of the animals were calculated. BF was measured using plethysmography as described previously (Novakova-jiresova et al., 2005). BF was defined as the number of breathes per minute. Temperature was measured using a DAS-7008/9 detector for s.c. injected electronic ID transponders, when animals were restrained (BMDS, Seaford, DE).

### Statistics

Mann-Whitney *U* test (two tailed) was used to test if the differences between two groups of the cotton rats tested for different parameters were significant. A *p* value of less than .05 was considered significant. *p* values less than .05, .01, and .001 are denoted by *, **, and ***, respectively. Graphs were plotted using GraphPad Prism 5 software (GraphPad Software, La Jolla, CA).

## Results

### Characterization of liquid and powder influenza vaccine formulations

The morphology of inulin, labeled, and unlabeled WIV powder formulations was examined by SEM. Particle size of powder formulations was determined by dispersion from RODOS and dry powder insufflator. Particle size of liquid formulation was determined by dispersion from microsprayer. Furthermore, the receptor binding capacity of SFD unlabeled WIV formulation was determined by hemagglutination assay.

#### Scanning electron microscopy

**Figure 1.**
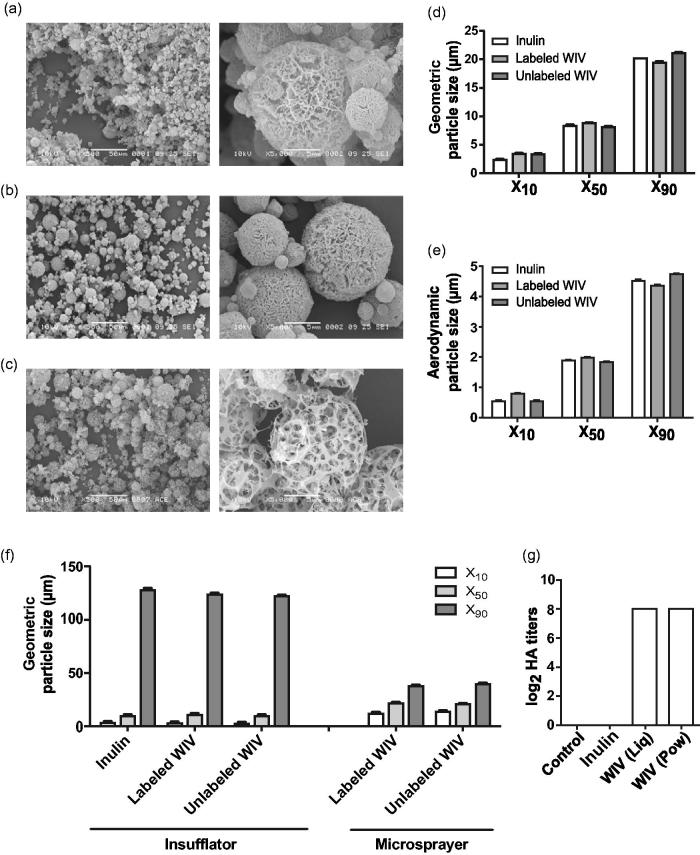
Characterization of liquid and powder formulations. Scanning electron microscopy (SEM) images of (a) inulin, (b) labeled WIV, and (c) unlabeled WIV powder formulations at 500× (left) and 5000× (right) magnification. (d) Geometric diameter of SFD inulin, labeled, and unlabeled powder particles using RODOS as the disperser. (e) Aerodynamic particle size of inulin, labeled, and unlabeled WIV formulations. (f) Particle size after dispersion of SFD powder particles using the dry powder insufflator and liquid WIV particles using the microsprayer. (g) Hemagglutination titers of control, reconstituted inulin, liquid and unlabeled powder WIV formulations. (*n* = 3); no differences were found among the triplicates within a group. Data are presented as average ± standard error of the mean (d–f; *n* = 6).

SEM indicated that the overall shape of inulin, labeled (HA: inulin 1:200) and unlabeled (HA: inulin 1:40) WIV powder particles was spherical (Figure 1(a–c)). All three powder formulations i.e. inulin, labeled, and unlabeled WIV particles had high porosity with an interconnected porous structure as found before for SFD powders (Saluja et al., 2010; Murugappan et al., 2013). Unlabeled WIV particles seemed to have higher porosity than labeled WIV particles (Figure 1(b,c)). This could be attributed to the increased HA: inulin ratio in the unlabeled WIV formulation.

#### Particle size of labeled and unlabeled vaccine powders

Laser diffraction analysis of labeled, unlabeled, and inulin powders revealed that 50% of the particles had a geometric particle size <9 µm and 90% of the particles had a size <22 µm. (Figure 1(d)) Geometric particle size was used to calculate aerodynamic particle size using the Equation (1):

(1) $$d_{\text{ae}}=d_{\text{e}}\sqrt{\left(\rho_{p}/\rho_{o}\text{ χ}\right)}$$

where *d*_ae_ is the aerodynamic diameter, *d*_e_ is the geometric particle size, *ρ*_p_ is the density of the particles (g/cm^3^), *ρ*_o_ is the unit density and χ is the dynamic shape factor.

The total solid content of the vaccine solution before spray freeze drying was 50 mg/mL which corresponds to a density (*ρ*_p_) of 0.05 g/cm^3^ of SFD powder particles, assuming no shrinkage or expansion during processing. Taking into account the geometric particle size (*d*_ae_), *ρ*_o_ (unit density: 1 g/cm^3^) and χ (one for spherical shaped particles), the aerodynamic particle size distribution was calculated. From the Equation (1) it was calculated that over 90 volume% of the inulin, labeled, and unlabeled WIV particles had aerodynamic particle size <5 µm, hence these particles were suitable for inhalation (Figure 1(e)). Overall, no differences in particle size were observed by the addition of fluorescent dye or an increase in HA content and the particle size was comparable to inulin only formulation.

#### Particle size upon dispersion from the Penn-Century insufflator and microsprayer

The dispersing capability of the insufflator was assessed by dispersion of SFD labeled WIV, unlabeled WIV, and inulin particles. The X_10_, X_50_ and X_90_ values for insufflator-dispersed particles are shown in Figure 1(f). The majority of the particles (X_90_) were found to be extremely large. It was found that 90% of the particles had sizes around 125 µm for all three powder formulations i.e. inulin, labeled,and unlabeled WIV. The dispersion of powder particles of size around 125 µm indicates that the insufflator was inefficient in de-agglomerating SFD powder particles even with 1 mL of air. However, when liquid vaccine formulations were dispersed from the microsprayer, the majority of the droplets (X_90_) had a geometric particle size of ∼40 µm (Figure 1(f)) which was about three-fold smaller than that from the insufflator (120 µm). Compared to insufflator-dispersed particles (3–140 µm), the particles dispersed by the microsprayer had a narrow size distribution (12–40 µm).

#### Hemagglutination titers

The biological activity of HA in unlabeled SFD formulation was assessed by determining its capacity to bind to the sialic acid receptors present on guinea pig RBC. The hemagglutination titers of reconstituted powder vaccine formulation were compared to those of the original untreated liquid WIV formulation. No difference in hemagglutination titers could be detected between reconstituted powder and the original liquid formulations, thus indicating that the hemagglutination activity was preserved after SFD for the unlabeled WIV formulation (Figure 1(g)).

### In vivo *deposition study*

**Figure 2.**
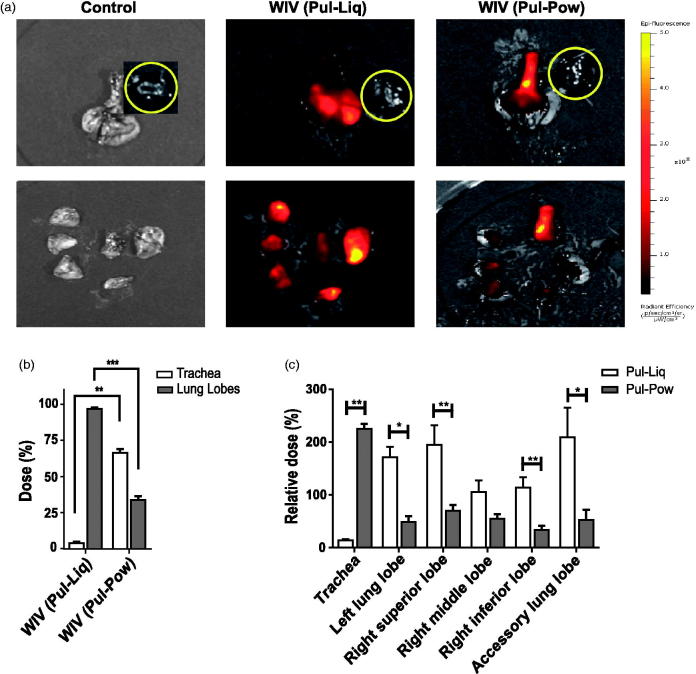
*In vivo* deposition of labeled liquid and powder WIV formulations. Cotton rats were vaccinated via the pulmonary route with labeled liquid and powder influenza vaccine formulations. Immediately after administration, animals were sacrificed and intact lungs were excised and later lungs were dissected into lung lobes for imaging. (a) Representative IVIS images of the intact (top) and dissected (bottom) lungs showing the deposition of liquid and powder WIV. Due to its proximity with the lungs, the heart (indicated by a yellow circle) was used as a negative control. (b) Percentage of the dose delivered in the trachea and five lung lobes (together) for liquid and powder WIV formulations relative to the total dose deposited in the respiratory tract. (c) Relative dose percentage in trachea and individual lung lobes for liquid and powder WIV formulations. Dose percentages for lung lobes were calculated as: (100-Dose deposition in trachea (%)). Relative dose percentages were determined as: dose % in trachea or lung lobe/Weight % of trachea or lung lobe out of the total lung weight. Data are presented as average ± standard error of the mean with levels of significance represented as *p < .05, **p < .01, ***p < .001.

To assess the distribution of labeled liquid and powder WIV formulations in cotton rats, imaging was performed on intact as well as dissected lungs using IVIS. Upon measurement, the total fluorescence intensity in trachea as well as lung lobes of the powder group was found to be ∼40% lower than in the liquid group. This implies an incomplete deposition of the powder dose in the respiratory tract with respect to liquid WIV formulation. Furthermore, IVIS images of whole lungs showed distribution of the liquid WIV formulation throughout the lungs whereas powders seemed to be deposited mainly in the trachea and central parts of lungs (Figure 2(a)). Upon dissection of the lungs into trachea and individual lung lobes, bright yellow fluorescent spots could be seen in lung lobes for the liquid WIV formulation and in the trachea for the powder WIV formulation. Further, upon quantification it was found that merely 4% of the liquid WIV formulation deposited in the respiratory tract remained within the trachea (Figure 2(b)) and thus that the majority of the liquid WIV formulation was distributed within the lung lobes (96%). However, for powders ∼66% of the WIV formulation that was deposited in the respiratory tract was found in the trachea and only ∼33% reached the central parts of the lung lobes (Figure 2(b)). Thus, the amount of WIV that deposited in the lungs was about 5 times higher for the liquid than for the powder formulation.

The distribution of liquid and powder WIV formulations was further assessed by calculating relative dose percentages in trachea and individual lung lobes of the cotton rats. A relative dose percentage of 100 would imply that the dose percentage deposited in that lung lobe or trachea is equal to its weight percentage in relation to the total lung weight. Liquid WIV formulations showed a relative dose of ∼13% in the trachea which further supports our conclusion of negligible deposition in the trachea (Figure 2(c)). Moreover, a relative dose of ∼200% was found in the left lung lobe, right superior, and accessory lung lobe which would imply twice the deposition of dose in these (right superior and accessory) lung lobes compared to the right middle and right inferior lung lobe (Figure 2(c)). The higher dose deposition in the left, right superior, and accessory lung lobes is in line with the bright yellow fluorescent spots observed in these lobes after excision of the whole lung (Figure 2(a)). However, for powders the trachea had a four-fold higher relative dose than the dose deposited in the lung lobes; a comparable but low relative dose percentage was found in all lung lobes (Figure 2(c)). This is further in agreement with dark yellow spots in the trachea and comparable dark red patches visible in the central parts of lung lobes of animals vaccinated with powder formulation.

### Systemic immune responses

**Figure 3.**
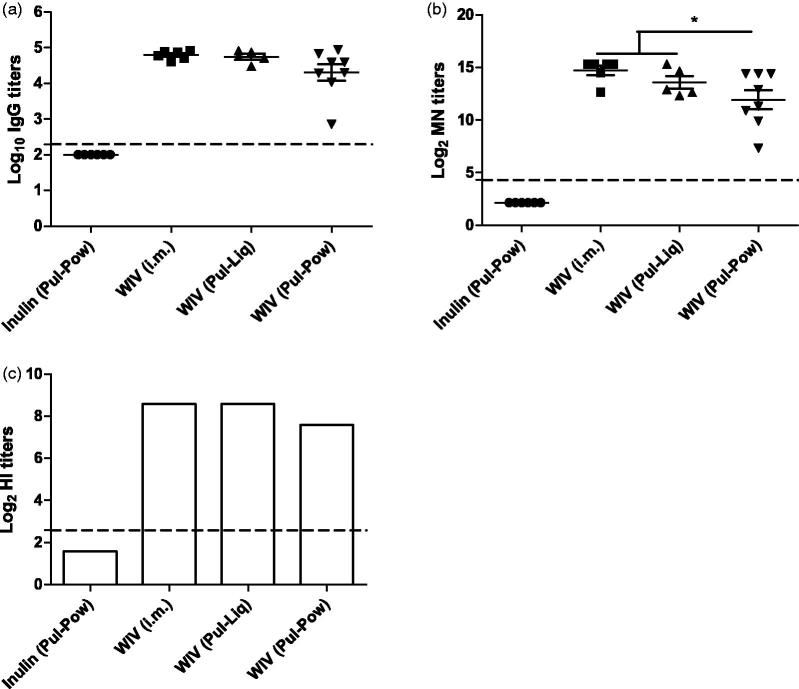
Systemic immune responses after pulmonary immunization. Cotton rats were vaccinated twice on day 0 and day 21 with liquid or powder formulations of WIV. Control cotton rats received SFD inulin powder via pulmonary route. Three weeks after the second vaccination (on the day of challenge), animals were bled and serum was used for determining systemic immune responses represented by (a) IgG titers (of individual cotton rats), (b) MN titers (of individual cotton rats), and (c) HI titers (sera pooled per experimental group). ELISA titers are represented as log_10_ titers, while MN and HI titers are represented as log_2_ titers with significance *p < .05. LoD is represented by dashed line at 2.3 for IgG, 4.32 for MN, and 2.59 for HI titers. Data are presented as average ± standard error of the mean [*n* = 6 for inulin (Pul-Pow) (•), *n* = 6 for WIV (i.m.) (▪), *n* = 5 for WIV (Pul-Liq) (▴), and *n* = 8 for WIV (Pul-Pow) (▾)].

To assess the systemic immune responses induced by liquid and powder WIV formulations upon pulmonary delivery, IgG ELISA, MN, and HI were performed using serum samples collected three weeks after the second vaccination. All cotton rats were tested seronegative for influenza specific antibodies before the start of the experiment (data not shown). After two vaccinations, animals from the inulin (Pul-Pow) group did not have any detectable serum IgG titers. Further, the IgG titers induced by pulmonary administered WIV were comparable to the titers induced by i.m. administered WIV (Figure 3(a)). Although IgG titers induced by WIV (Pul-Pow) were lower than the IgG titers induced by WIV (Pul-Liq), the difference was not significant. The functional assays, i.e. MN (Figure 3(b)) and HI (Figure 3(c)) assay, showed that the antibodies generated by all three vaccine formulations had the potential to neutralize virus and inhibit virus hemagglutination in an *in-vitro* setting. WIV (Pul-Liq) induced significantly higher MN titers than WIV (Pul-Pow). Also, the HI titers of WIV (Pul-Liq) were higher than WIV (Pul-Pow). Thus, pulmonary delivered liquid WIV was found to be slightly more potent in inducing functional systemic immune responses than the pulmonary delivered powder WIV formulation.

#### Mucosal immune responses

**Figure 4.**
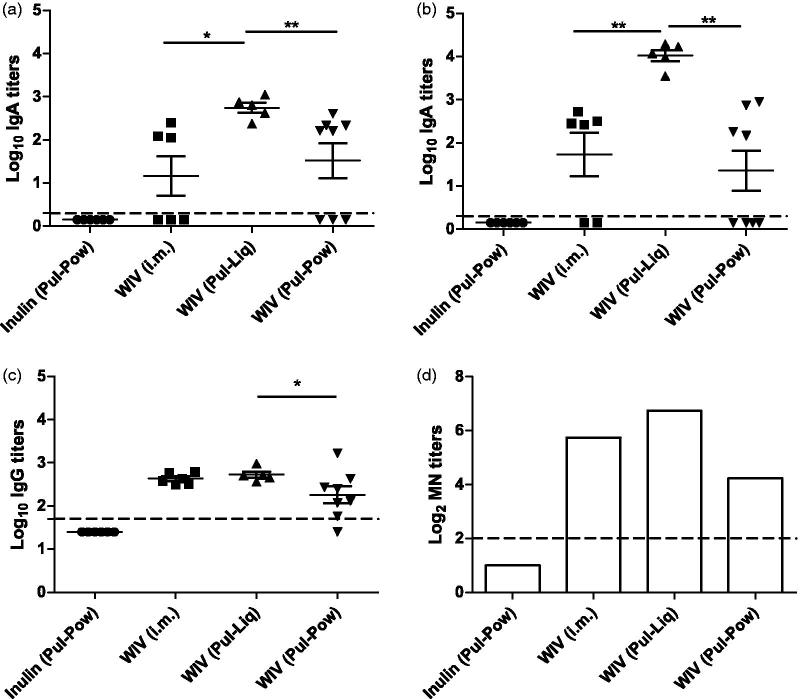
Mucosal immune responses after pulmonary immunization. Cotton rats were vaccinated twice followed by a homologous challenge with 10^7^ TCID50/ animal of A/Cal/2009. One day post challenge some animals were sacrificed, nasal, and lung washes were collected. Mucosal immune responses are represented as (a) Nose IgA titers,(b) Lung IgA titers, (c) Lung IgG titers, and (d) MN titers from lung washes pooled per group. Titers are represented as log_10_ titers along with the significance indicated as *p < .05 and **p < .01. LoD is represented by a dashed line at 0.3 for both nose and lung IgA, while at 1.7 for lung IgG, and at 2 for lung MN titers. Data are presented as average ± standard error of the mean [*n* = 6 for inulin (Pul-Pow) (•), *n* = 6 for WIV (i.m.) (▪), *n* = 5 for WIV (Pul-Liq) (▴), and *n* = 8 for WIV (Pul-Pow) (▾)].

To assess the mucosal immune responses induced by pulmonary delivered liquid and powder influenza vaccine formulations, IgA titers were determined using nasal and lung washes taken from animals sacrificed one day post challenge. Along with IgA, IgG, and MN titers were also determined from lung washes. Animals administered with inulin (Pul-Pow) were negative for mucosal antibodies. WIV (Pul-Liq) induced nasal and lung IgA in all animals and IgA titers were significantly higher in this group than WIV (Pul-Pow; Figure 4(a,b)) respectively. Some cotton rats of the WIV (Pul-Pow) group were found to be non-responders for nasal (3/8) and lung IgA (4/8), while some of the animals vaccinated via i.m. route were found to have IgA in their nose (3/6) and lungs (4/6). However, the titers generated by WIV (i.m.) were found to be significantly lower than the titers generated by WIV (Pul-Liq) group in which all animals were responders. After pulmonary immunization, the detection of nasal antibodies can possibly be attributed to the migration of antigen specific B cells from the site of induction (lungs) to distant mucosal site (nose) (McDermott & Bienenstock, 1979; Neutra et al., 1996). Further, WIV (i.m.) and WIV (Pul-Liq) formulations induced comparable IgG antibody titers in lungs (Figure 4(c)). Lung IgG titers induced by WIV (Pul-Pow) were significantly lower than IgG titers induced by WIV (Pul-Liq). Moreover, mucosal antibodies in the lung washes were found to neutralize the virus *in vitro* (Figure 4(d)) and the trend for these MN titers (higher for liquids than for powders) was similar to the trend observed in ELISA titers (Figure 4(b,c)). Thus, pulmonary delivered liquid WIV was found to be more immunogenic in inducing mucosal immune responses than the pulmonary delivered powder WIV formulation.

### Lung viral load

**Figure 5.**
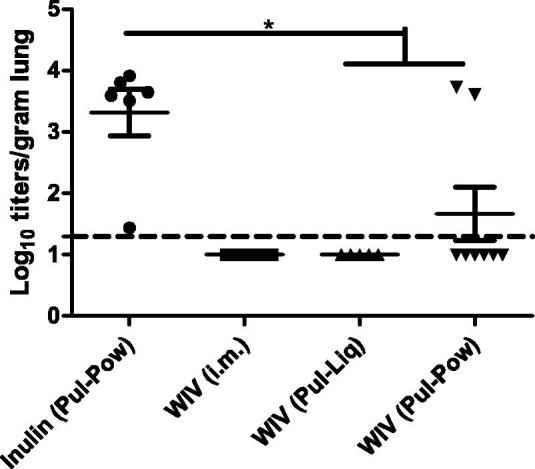
Effect of pulmonary vaccination on lung virus titers upon live virus challenge. Three weeks after the second vaccination cotton rats were challenged with 10^7^ TCID50/ animal of A/Cal/2009 virus. One day post challenge some animals were sacrificed and their lungs were homogenized to determine virus load. Virus titers are represented as log_10_ titers per gram of lung and significant differences between titers of different groups are represented as *p < .05. LoD is represented by a dashed line at 1.3. Data are presented as average ± standard error of the mean [*n* = 6 for inulin (Pul-Pow) (•), *n* = 6 for WIV (i.m.) (▪), *n* = 5 for WIV (Pul-Liq) (▴), and *n* = 8 for WIV (Pul-Pow) (▾)].

To assess if the pulmonary delivered influenza vaccine formulations could reduce the viral load in the lungs after challenge, virus titration was done using the lungs of animals sacrificed on day one post challenge (Figure 5). It has been shown before that lung virus replication peaks one day after challenge in cotton rats (Ottolini et al., 2005; Straight et al., 2006). In line with that, we could also detect virus in the lungs of animals administered with inulin (Pul-Pow) one day post-challenge (with mean titer of about 10^3.3^), indicating successful infection. No detectable virus titers were found in the lungs of the cotton rats vaccinated with WIV (i.m.) and WIV (Pul-Liq). Also, six out of eight animals immunized with WIV (Pul-Pow) did not have any detectable virus in their lungs. However, in two WIV (Pul-Pow) immunized animals, virus could be detected in the lungs. Interestingly, these two animals were the ones with no nasal and lung IgA antibodies and one of the two also had low serum IgG titers. Overall, the majority of the animals vaccinated with WIV via the pulmonary route did not have detectable virus in their lungs.

### Clinical symptoms

**Figure 6.**
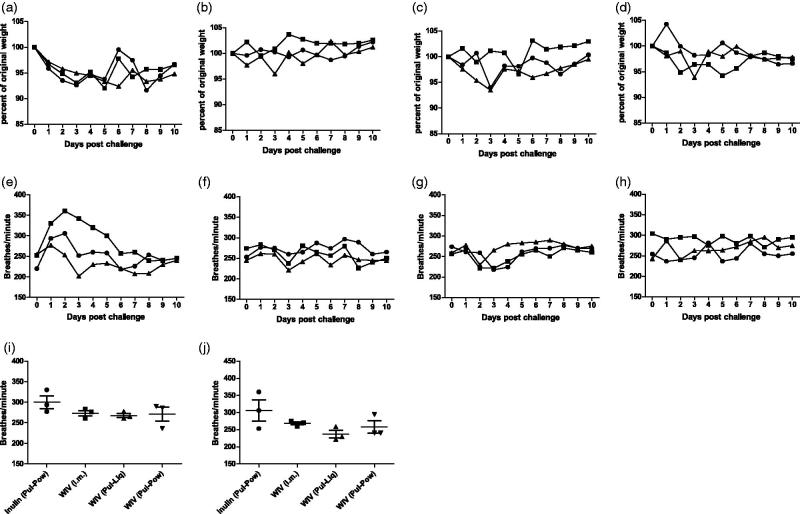
Effect of pulmonary vaccination on clinical symptoms after live influenza virus challenge. Upon vaccination and homologous live virus challenge, cotton rats were followed for 10 days for assessment of weight loss (a) Inulin (Pul-Pow), (b) WIV (i.m.), (c) WIV (Pul-Liq), and (d) WIV (Pul-Pow). Also, change in the BF was evaluated for all the animals that were followed (e) Inulin (Pul-Pow), (f) WIV (i.m.), (g) WIV (Pul-Liq), and (h) WIV (Pul-Pow). Day wise BF was plotted for (i) day 1 and (j) day 2 post challenge for animals in each group. Data [(i),( j)] are presented as average ± standard error of the mean . For a to h, each line represents 1 animal. For each figure, •: animal 1; ▪: animal 2; ▴: animal 3. For i and j [*n* = 3 for inulin (Pul-Pow) (•), *n* = 3 for WIV (i.m.) (▪), *n* = 3 for WIV (Pul-Liq) (▴), and *n* = 3 for WIV (Pul-Pow)(▾)].

To assess the protection conferred by pulmonary delivered WIV formulations, animals were followed for ten days after challenge to evaluate the following clinical symptoms, i.e. weight loss, increase in BF, and drop in body temperature. Animals administered with inulin (Pul-Pow) showed a trend towards decrease in body weight after challenge, although it was not substantial (Figure 6(a)). No or little weight loss was observed for animals vaccinated with WIV (i.m.) (Figure 6(b)), WIV (Pul-Liq) (Figure 6(c)), or WIV (Pul-Pow) (Figure 6(d)). Overall, live virus challenge did not have much influence on weight in any of the animals.

Animals administered with inulin (Pul-Pow) showed an increase in BF after challenge for two days (Figure 6(e)) after which the BF gradually decreased. Animals vaccinated with WIV (i.m.) (Figure 6(f)), WIV (Pul-Liq) (Figure 6(g)) or WIV (Pul-Pow) (Figure 6(h)) showed no or very little increase in the BF over the period of 10 days. Days-wise BF was plotted for animals from all the groups for day 1 (Figure 6(i)) and day 2 (Figure 6(j)). A difference of ∼30 breathes/minute for day one and ∼60 breathes/minute for day two was observed between the animals from the inulin and the pulmonary WIV groups. Hence, pulmonary administered WIV formulations protected cottons rats from infection induced increase in breathing. There was no effect of challenge on the temperature in non-vaccinated as well as vaccinated animals (Supplementary Figure 1).

## Discussion

In the current study, we showed that cotton rats can be successfully vaccinated against influenza via the pulmonary route. Further, it was demonstrated that pulmonary administered liquid WIV was predominantly deposited throughout the lungs while powder WIV was deposited mainly in the trachea, indicating clear differences in sites of deposition. Interestingly, a vaccination-challenge study revealed differences in particularly mucosal immune responses, but had only minor impact on protection upon live virus challenge.

Liquid and powder formulations were deposited in different parts of the respiratory tract, but both these formulations induced potent systemic immune responses. Besides systemic immune responses, an important aspect of mucosal vaccination is the induction of IgA antibody at mucosal sites (Srivastava et al., 2015; Kim & Jang, 2017). Even though in some previous studies with influenza vaccines, cotton rats were vaccinated via the i.n. route, mucosal immunity was not evaluated (Straight et al., 2008; Crosby et al., 2017). Here for the first time we show that, upon pulmonary influenza vaccination, mucosal antibody (IgA and IgG) responses along with systemic immune responses can be achieved in this animal model. Overall, WIV (Pul-Liq) induced better immune responses than WIV (Pul-Pow) in particular in the respiratory tract.

Although differences in immune responses were obvious between animals vaccinated with liquid and powder formulations, protection with respect to prevention of clinical symptoms conferred by both these formulations was rather similar. Previous studies in mice have shown that mucosal antibodies (IgA and IgG) contribute to virus neutralization *in vivo* (Onodera et al., 2012). In line with this, we also observed that mucosal antibodies induced upon pulmonary vaccination could neutralize the challenge virus *in vitro*, possibly indicating their role in the clearance of the virus at the portal of entry. The two animals having lung virus titers from the WIV (Pul-Pow) group had no lung and nasal IgA. Also, one of these two animals had no lung IgG while the other had low lung IgG titers. The remaining two non-responders for lung IgA from the WIV (Pul-Pow) group cleared virus from the lungs, which could be attributed to the presence of IgG antibodies in their lungs. This further supports the role of mucosal antibodies (IgA and IgG) in virus neutralization.

Of the clinical symptoms assessed in the vaccinated and challenged animals, BF was earlier found to be the most sensitive parameter of infection. It is known that virus replication in the respiratory tract leads to epithelial damage, which in turn causes difficulties in breathing in cotton rats (Trias et al., 2009). Most likely this causes an increase in BF of infected animals. However, cotton rats pulmonary administered with WIV (liquid or powder) showed little or no increase in BF compared to those pulmonary administered with inulin indicating that protection from virus replication in the lungs results in normal breathing. Further, we found little effect of H1N1pdm challenge on weight and no effect on the temperature of cotton rats administered with inulin (Pul-Pow). This is also consistent with what has been observed previously; infection with H1N1pdm influenza virus, in contrast to infection with other influenza virus strains, does not affect the weight and temperature of cotton rats (Blanco et al., 2013). Overall, no differences were observed in the protective efficacy (lung virus titers and BF) of WIV (Pul-Liq) and WIV (Pul-Pow) formulations; though a difference in deposition site was obvious.

In this study, the tip of the insufflator was placed at the bifurcation of trachea i.e. carina, thus whole lung deposition might be expected. However, the introduction of 1 mL of air to an already inflated lung caused a return flow of air containing powder particles. This return flow further most likely caused the majority of the particles to be deposited in the trachea. Moreover, it was visually observed that a fraction of the particles were even exhaled resulting in incomplete dosing. In addition, it was noticed that a portion of the powder remained in the insufflator after three puffs further reducing the delivered dose. The incomplete dosing of the powder formulation was further evident from the detection of reduced fluorescence intensity in the respiratory tract of cotton rats compared to the fluorescence intensity in liquid vaccinated group. Furthermore, as also reported by Murugappan et al. (2013) the insufflator was not able to break up agglomerates of the SFD powder into primary particles as 90 volume% of the dispersed particles had a geometric particle size of ∼130 µm. This particle size corresponds to an aerodynamic diameter of 29 µm, which by far exceeds the required size range of 1–5 µm for whole lung deposition. Thus, pulmonary delivery of SFD powder particles to cotton rats using the insufflator results in incomplete dosing and very limited whole lung deposition. Therefore, we utilized this inefficient de-agglomeration property of the insufflator to achieve powder deposition in trachea. Hence, we would like to emphasize this fact that, the powder deposition in trachea was due to the limitations of the pre-clinical device available for administration rather than the characteristics or the quality of formulation itself.

By contrast, no air is used during administration of the liquid vaccine formulation with the microsprayer. Therefore, for the liquid formulation, no substantial return flow of air is expected. Furthermore, no substantial amount of liquid remained in the microsprayer. Thus, dosing was complete or nearly complete. The microsprayer produced droplets with a geometric size of 12–40 µm. As the density of the droplets was around 1 g/cm^3^, the aerodynamic diameter was similar to the geometric size and thus also too large for whole lung deposition. Apparently, a volume of 50 µL was sufficient for a ‘dripping down effect’ to reach the alveolar regions of the lung. Thus, complete dosing of the liquid vaccine led to the induction of better immune responses than powder vaccine which had the incomplete dosing problems due to the inefficient insufflator and return flow due to the breathing of the animals.

In order to target different parts of the respiratory tract, Minne et al. (2007) administered liquid formulations with different dose volumes and also positioned mice at different angles during administration. They reported deep lung deposition of influenza vaccine to be superior to deposition in the upper or central airways. It was speculated that an improved residence time of influenza antigen when targeted deep into the lungs (due to reduced mucociliary clearance) promotes a better immune response. However, our study shows that even though deposited in the trachea, WIV powder formulation had comparable protective efficacy as WIV liquid formulation deposited deep in the lungs. This could be due to the use of inulin in the WIV powder formulation; inulin might enhance the viscosity at the site of dissolution and thus the problem of mucociliary clearance might be countered. This might in turn increase the residence time and thereby uptake of WIV antigen by antigen presenting cells (APCs) which are abundantly present both in the trachea and in the lungs of rodents (Sertl et al., 1986; Holt et al., 1988). Also, human trachea and lungs both contain a large number of dendritic cells that can take up antigens and transport them to the draining lymph nodes (Tschernig et al., 2001, 2006; Lambrecht & Hammad, 2012; Neyt & Lambrecht, 2013). Thus, both these sites, trachea as well as lungs, could be favorable for influenza vaccine uptake to elicit a potent systemic and local immune response. Hence, the observed differences in immune responses could be predominately due to the incomplete dosing of the WIV powder as APCs are present in both trachea as well as lungs.

The current study demonstrates that the immune responses generated by influenza vaccine formulations deposited either in the trachea or deep lung can be potent enough to provide protection against live virus challenge. Since the site of deposition seems less important for the success of pulmonary influenza vaccination, we believe that a dry powder influenza vaccine formulation is preferable to a liquid formulation because of the following reasons; (i) Availability of better clinical devices for dispersion of powders: though in this study we showed that the deposition of WIV (Pul-Pow) was predominantly in the trachea of cotton rats, deposition over the whole lungs might still be beneficial. Better lung deposition is expected in a clinical setting because better powder delivery devices for humans are available (Twincer, Torus). These devices work on the principle of active inhalation by the individual thereby preventing exhalation of the powder by return flow (de Boer et al., 2006; Tonnis et al., 2013; Tomar et al., 2016). Moreover, in a clinical setting the use of a dry powder inhaler with low retention can further resolve dosing issues associated with Penn-insufflator; (ii) Long term stability: previous studies have shown that by using appropriate formulation strategies, dry powder influenza vaccine formulations can be prepared with long term stability at room temperature (Geeraedts et al., 2010; Saluja et al., 2010; Murugappan et al., 2013; Flood et al., 2016; Lovalenti et al., 2016). This alleviates the need of cold chain for powder influenza vaccines thus making it suitable for stockpiling, which is of great importance during pandemics (Tomar et al., 2016). Hence, in a clinical setting pulmonary delivery of inhalable dry powder influenza vaccine formulations would be advantageous over liquid formulations.

## Conclusion

In conclusion, we demonstrate that cotton rats can be used as a model for pulmonary influenza vaccination. Sites of deposition of liquid and powder influenza vaccine formulations were clearly different upon pulmonary delivery. However, the deposition site did not have a major impact on the protective efficacy of these formulations in cotton rats.

## Supplementary Material

IDRD_Hinrichs_et_al-Supplemental_Content.eps
